# Circulating Transforming Growth Factor-β and Aortic Dilation in Patients with Repaired Congenital Heart Disease

**DOI:** 10.1038/s41598-018-36458-1

**Published:** 2019-01-17

**Authors:** Yiu-fai Cheung, Pak-Cheong Chow, Edwina Kam-fung So, Koon-wing Chan

**Affiliations:** Department of Paediatrics and Adolescent Medicine, Queen Mary Hospital, The University of Hong Kong, Pok Fu Lam, China

## Abstract

This study determined the circulating levels of TGF-β1 and its association with aortic dilation and elastic properties in congenital heart patients. Forty-six patients after tetralogy of Fallot (TOF) repair, 21 patients post arterial switch and 15 patients post atrial switch for transposition of the great arteries (TGA), 27 patients post Fontan procedure, and 36 controls were studied. Aortic dimensions and elastic properties and ventricular function were assessed by echocardiography. Serum TGF-β1, metalloproteinase (MMP)-2 and MMP-9 levels were quantified. Compared with controls, all groups of patients had significantly larger ascending aortic dimensions and worse elastic properties (all p < 0.05). Aortic stiffness correlated positively with sinus dimension (r = 0.48, p < 0.001) and negatively with indices of ventricular deformation (all p < 0.001). Patients with repaired TOF had significantly higher levels of TGF-β1 (p = 0.005), MMP-2 (p = 0.001) and MMP-9 (p < 0.001) than controls, while patients after atrial switch operation (p = 0.034) and Fontan procedures (p < 0.001) had higher MMP-2 levels. In patients as a group, circulating TGF-β1 levels correlated with MMP-9 (r = 0.44, p < 0.001) and aortic sinus dimension (r = 0.22, p = 0.035). In conclusion, increased circulating TGF-β1, MMP-2, and MMP-9 levels were found in patients with repaired TOF, and increased circulating MMP-2 levels were also evident in patients after atrial switch operation and Fontan procedure.

## Introduction

Progressive dilation of the aortic root has become a growing concern in the adolescent and adult congenital heart population, and it is associated with aortic regurgitation, stiffening, and dissection^1–3^. While alteration of haemodynamics has been hypothesized to cause aortic dilation, there is evidence of a primary aortopathy^4^. Histological abnormalities of the aortic root as characterized by medionecrosis, fibrosis, and fragmentation of elastic fibres have been found in different congenital heart conditions including tetralogy of Fallot (TOF) with or without pulmonary atresia, complete transposition of the great arteries (TGA), and functionally univentricular hearts^4,5^. Functional alteration of aortic elastic properties has further been demonstrated in these patients^6–9^. The interplay between functional and structural abnormalities may result in a feedback loop that leads to progressive aortic dilation. A better understanding of vascular remodeling in these congenital heart patients may shed light on the management of progressive aortic root dilation and its complications.

The importance of the transforming growth factor-beta (TGF-β) superfamily of cytokine in vascular remodeling is increasingly recognized. In aortic aneurysms related to bicuspid aortic valves, the altered TGF-β1 signaling activity may increase production of matrix metalloproteinase (MMP) −2 and −9 and lead to matrix degradation^10^. In a mice model of Marfan syndrome, deficiency of normal fibrillin may lead to the release of sequestered latent form of TGF-β1, increasing its activity^11^. Importantly, increased circulating levels of TGF-β1 found in patients with Marfan syndrome have been shown to be associated with the magnitude of aortic root dilation, the occurrence of cardiovascular events including aortic dissection, and the need for aortic root replacement^12,13^. Interestingly, overexpression of TGF-β1 has also been reported in the ascending aorta of congenital heart disease^14^. Nonetheless, the clinical relevance of circulating levels of TGF-β1 in congenital heart patients who are at risk of aortic root dilation is unknown.

Given the increasing evidence of involvement of TGF-β1 signaling in aortic remodeling, we hypothesized that circulating TGF-β1 levels are altered in patients with repaired congenital heart disease at risk of ascending aortopathy. In this study, we aimed to determine the circulating levels of TGF-β1 and to explore their associations with aortic dilation and aortic elastic properties in adolescents and young adults after TOF repair, arterial and atrial switch operations for complete TGA, and Fontan procedure for univentricular hearts.

## Methods

### Subjects

The inclusion criteria of the study was adolescent and adult congenital heart patients who had undergone surgical repair of TOF, arterial switch operation and atrial switch operation for complete TGA, and Fontan procedure for functionally univentricular hearts. They were consecutively recruited from our congenital heart clinic. The following clinical data were retrieved from the case notes: age at operation, follow-up duration, associated cardiac lesions, and residual postoperative cardiac lesions. Healthy subjects, who are staff members of the hospital and their relatives and friends, with no known cardiovascular disease were recruited as controls. None of the controls are family members of patients. This study was approved by the Institutional Review Board of the University of Hong Kong/Hospital Authority Hong Kong West Cluster, Hong Kong, and all of the methods as described were performed in accordance with the approved guidelines and regulations. All of the adult subjects and parents of minors gave written informed consent.

The body weight and height of the subjects were measured and the body mass index and body surface area were calculated accordingly. All of the subjects rest for at least 15 minutes before vascular and echocardiographic assessments as described below.

### Assessment of aortic size and mechanics

Vascular and echocardiographic assessments were performed using the Vivid 7 ultrasound machine (GE Medical System, Horten, Norway). The average values of the echocardiographic indices from three cardiac cycles were obtained.

The size of the aortic root was measured at diastole from the parasternal long-axis view at four levels: annulus, sinus of Valsalva, sinotubular junction, and proximal ascending aorta. Severity of aortic regurgitation was assessed semi-quantitatively by colour Doppler flow mapping.

Elastic properties of the ascending aorta were determined using M-mode echocardiography performed from the parasternal long-axis view^8,15^. Systolic (AoS) and diastolic (AoD) dimensions of the proximal ascending aorta were measured, while systolic (SBP) and diastolic (DBP) blood pressures of the right upper arm were obtained using an automated oscillometric device (Dinamap, Critikon, Inc., Tampa, Florida). The following parameters of aortic elastic properties were calculated: (i) strain = (AoS-AoD)/AoD x 100%, (ii) distensibility = [2 × (AoS-AoD)])/[AoDx(SBP-DBP)], and (iii) stiffness index = ln(SBP/DBP)/[(AoS-AoD)/AoD].

For assessment of regional arterial stiffness, the brachial-ankle pulse wave velocity was determined using an automated device (VP-2000; Colin Medical Technology, Komaki, Japan)^6^. Briefly, oscillometric cuffs were wrapped around the brachium and ankles to register the pressure pulse and the right and left brachial-ankle pulse wave velocities were calculated by dividing the pulse wave traveling distance by the transit time. The traveling distance was determined automatically based on the height of the subject.

### Echocardiographic assessment

To assess the potential impact of altered aortic elastic properties on systemic ventricular function, echocardiographic assessment of the systemic ventricle was determined by tissue Doppler imaging and two-dimensional speckle tracking echocardiography.

Tissue Doppler imaging was performed with the sample volume positioned at the basal systemic ventricular free wall-valvar annular junction to obtain the following indices: peak systolic (*s*) myocardial tissue velocity, peak early (*e*) and late (*a*) diastolic myocardial tissue velocities, *e*/*a* ratio, and myocardial acceleration during isovolumic contraction. For Fontan patients, the average of the right- and left-sided annular tissue Doppler velocities was used for analysis.

Global longitudinal and circumferential strain and strain rate of the systemic ventricle were assessed by two-dimensional speckle tracking echocardiography as reported previously^16^. The apical four-chamber view was acquired for assessment of global longitudinal strain and strain rate, while the short-axis view at the mid-ventricular level was acquired for assessment of circumferential strain and strain rate using the EchoPAC software (GE Medical Systems, Horten, Norway).

### Blood investigations

Blood samples were collected by venepuncture and serum samples were stored at −80 °C until assay. Total TGF-β1 level was measured by commercially available assay (TGF-beta 1 Quantikine immunoassay, R&D Systems, Minneapolis, Minnesota). Serum levels of MMP-2 and MMP-9 were also determined using enzyme-linked immunosorbent assays (Quantikine immunoassay, R&D Systems, Minneapolis, Minnesota). These assays measured respectively the total MMP-2 levels and total pro- and active MMP-9 levels.

### Statistical analysis

All data are presented as mean ± SD. The aortic dimensions were measured by a single investigator (EKS) to avoid inter-observer bias. Comparisons of cardiovascular indices and circulating levels of TGF-β1, MMP-2, MMP-9 among different groups were performed using simple analysis of variance. Comparisons of cardiovascular and blood indices between controls and each of the patient cohorts were performed using unpaired Student’s t test. Relationships between TGF-β1 and MMP levels and those between TGF-β1 and aortic dimensions and elastic properties were assessed by Pearson correlation analysis. Multivariate analysis that takes also into account of the age, sex, type of congenital heart disease, aortic sinus dimension and stiffness was performed to identify significant correlates of the circulating levels of TGF-β1. A p value of <0.05 is considered statistically significant. All statistical analyses were performed using SPSS Statistics 19 (SPSS Inc, Chicago, IL, USA).

## Results

### Subjects

Table 1 summarizes the demographic and clinical data of the 145 subjects recruited. A total of 109 (66 males) patients and 36 (16 males) controls were studied. Of the 109 patients, 46 had undergone surgical repair of TOF (43 had TOF with pulmonary stenosis, and 3 had TOF with pulmonary atresia), 21 had undergone arterial switch operation for TGA, 15 had undergone atrial switch operation for TGA, and 27 had undergone Fontan operation. As a group, patients were followed up for 24.0 ± 5.7 years. Their residual postoperative cardiac lesions are shown in Table 1.

**Table 1:** Demographic and clinical data.

	Repaired TOF (n = 46)	TGA post ASO (n = 21)	TGA post atrial switch (n = 15)	Fontan (n = 27)	Controls (n = 36)
***Demographic data***					
Age at study (years)	29.6 ± 7.2	21.9 ± 2.2	30.6 ± 4.5*	26.1 ± 4.6	26.9 ± 7.4
Sex (male: female)	23:23	12:9	13:2	18:9	16:20
Weight (kg)	59.5 ± 13.2	59.9 ± 11.0	61.4 ± 8.7	55.1 ± 13.2	59.9 ± 14.5
Height (m)	1.6 ± 0.1	1.7 ± 0.1	1.7 ± 0.0	1.6 ± 0.1	1.6 ± 0.1
BMI (kg/m^2^)	22.2 ± 4.0	22.1 ± 4.3	21.8 ± 2.9	20.4 ± 3.8	21.9 ± 3.6
BSA (m^2^)	1.6 ± 0.2	1.7 ± 0.1	1.7 ± 0.1	1.6 ± 0.2	1.6 ± 0.2
SBP (mmHg)	113 ± 10	119 ± 12*	120 ± 9*	110 ± 13	107 ± 10
DBP (mmHg)	65 ± 5	68 ± 8*	69 ± 5	66 ± 5*	62 ± 6
***Surgery***					
Age at surgery (years)	4.22 ± 2.65	0.04 ± 0.02	1.32 ± 1.17	4.79 ± 3.28	
Duration after repair (years)	25.1 ± 6.6	21.5 ± 2.3	29.1 ± 3.9	20.9 ± 3.7	
***Valvar regurgitation***					
**Systemic AV valve**					
Mild	5	2	5	10	
Moderate	0	0	0	0	
**Subpulmonary AV valve**					
Mild	35	4	11	—	
Moderate	4	0	3	—	
**Pulmonary valve**					
Mild	12	3	5	—	
Moderate	13	1	0	—	
Severe	12	0	0	—	
**Aortic valve**					
Mild	29	14	6	12	
Moderate	1	2	0	1	
***Residual pulmonary arterial stenosis***					
MPA	4	1	0	—	
RPA	4	3	0	—	
LPA	5	2	0	—	
***Residual left-to-right shunt***					
VSD	1	0	0	0	

Abbreviations: AV, atrioventricular; BMI, body mass index; BSA, body surface area; DBP, diastolic blood pressure.

LPA, left pulmonary artery; MPA, main pulmonary artery; RPA, right pulmonary artery; SBP, systolic blood pressure.

VSD, ventricular septal defect.

*p < 0.05 compared with controls.

Compared with controls, patients as a group had similar body weight (p = 0.46), body height (p = 0.46), body mass index (p = 0.35), and body surface area (p = 0.45).

### Aortic root dimensions and elastic properties

Table 2 shows the dimensions of the aortic root in all patients and controls. Compared with controls, all of the four groups of patients had significantly larger aortic annulus, sinus of Valsalva, sino-tubular junction and proximal ascending aorta (all p < 0.05). The average intra- and interobserver variabilities in the measurement of aortic root dimensions were 4.8% and 5.2%, respectively.

**Table 2:** Aortic root dimensions.

	Repaired TOF (n = 44^#^)	TGA post ASO (n = 21)	TGA post atrial switch (n = 14^#^)	Fontan (n = 27)	Controls (n = 36)
Annulus (cm)	2.21 ± 0.41*	2.45 ± 0.36*	2.40 ± 0.34*	2.33 ± 0.52*	1.90 ± 0.23
Sinus of Valsalva (cm)	3.36 ± 0.37*	3.38 ± 0.38*	3.32 ± 0.33*	3.32 ± 0.52*	2.63 ± 0.35
Sino-tubular junction (cm)	2.83 ± 0.43*	2.96 ± 0.45*	2.66 ± 0.35*	2.66 ± 0.50*	2.05 ± 0.36
Ascending aorta (cm)	2.80 ± 0.44*	3.04 ± 0.48*	2.76 ± 0.41*	2.68 ± 0.52*	2.11 ± 0.39

*p < 0.05 compared with controls.

^#^A total of three patients had suboptimal images for measurement of root dimensions.

The prevalence of aortic regurgitation was 76% and 65% in patients after arterial switch operation and repair of TOF, respectively. On the other hand, the prevalence was lower in Fontan patients and in patients after atrial switch operation at 48% and 40%, respectively (Table 1).

**Figure 1:**
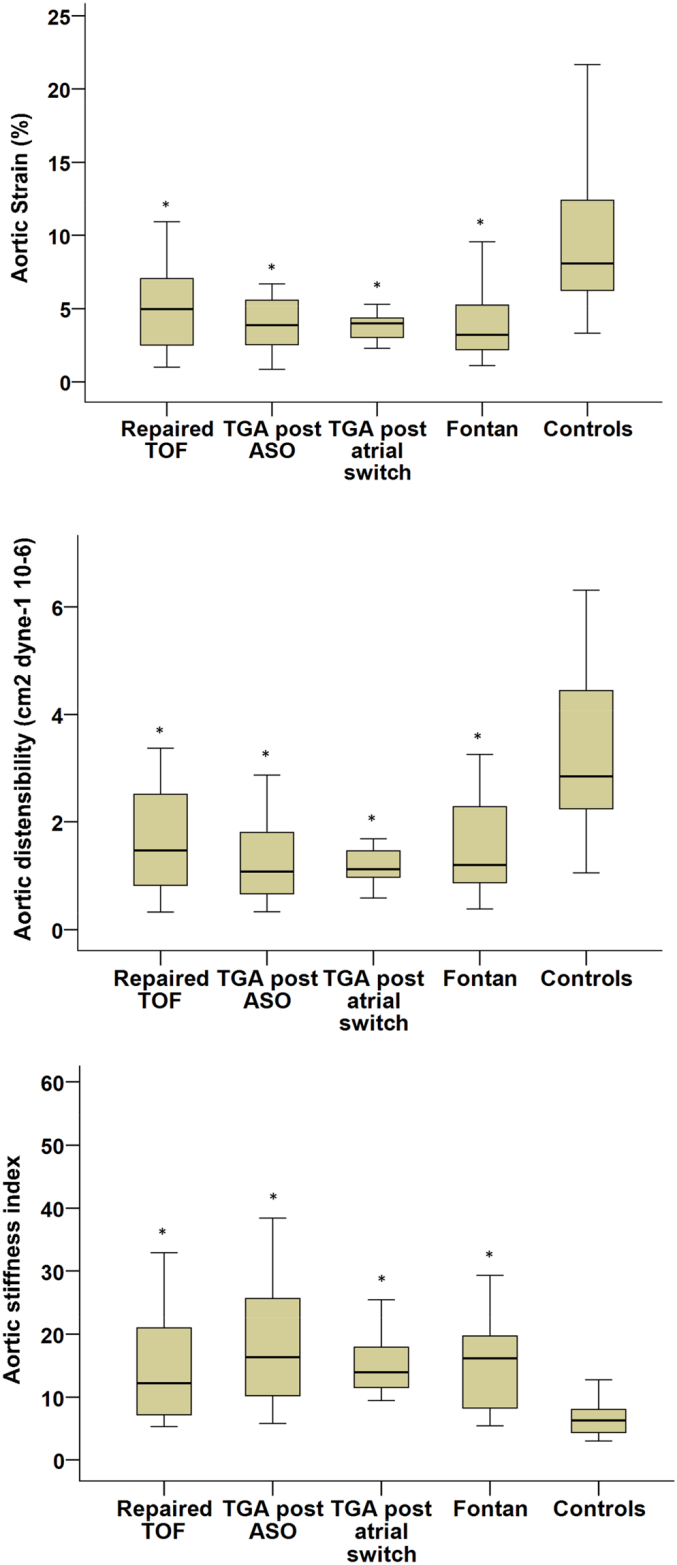
Aortic elastic properties in patients and controls (*p < 0.05 compared with controls). ASO, arterial switch operation, TGA, transposition of the great arteries, TOF, tetralogy of Fallot.

For the aortic elastic properties, all of the four patient groups had significantly lower aortic strain (all p < 0.001) and aortic distensibility (all p < 0.001) and greater aortic stiffness index (all p < 0.001) than controls (Fig. 1). On the other hand, the average right and left brachial-ankle pulse wave velocity, which reflects the composite stiffness of aorta and peripheral arteries, was similar between all four groups of patients (repaired TOF, 11.2 ± 1.6 m/s, TGA post arterial switch, 10.5 ± 1.6 m/s, TGA post atrial switch 11.2 ± 1.6 m/s, Fontan, 11.2 ± 1.8 m/s) and controls (10.9 ± 1.3 m/s) (all p > 0.05).

For the entire cohort, aortic strain (r = −0.50, p < 0.001), distensibility (r = −0.51, p < 0.001), and stiffness index (r = 0.48, p < 0.001) correlated significantly with aortic sinus dimension.

### Echocardiographic parameters of cardiac function

Table 3 summarizes the echocardiographic parameters of ventricular function in all patients and controls. Doppler assessment of systemic ventricular inflow revealed significantly lower E velocity and E/A ratio in Fontan patients compared with controls (both p < 0.001). Tissue Doppler echocardiography revealed worse systolic and diastolic function of the systemic ventricle in patients compared with controls as demonstrated by their significantly lower *s* and *e* velocities and systemic ventricular IVA (all p < 0.01).

**Table 3:** Echocardiographic indices of systemic ventricular function.

	Repaired TOF (n = 46)	TGA post ASO (n = 21)	TGA post atrial switch (n = 15)	Fontan (n = 27)	Controls (n = 36)
***Systemic atrioventricular inflow doppler indices***					
E (cm/s)	93.3 ± 22.0	98.6 ± 21.8	91.8 ± 21.6	64.1 ± 13.1*	97.3 ± 20.2
A (cm/s)	47.5 ± 14.1	44.8 ± 14.2	49.7 ± 18.4	46.3 ± 9.3	46.4 ± 9.9
E/A ratio	2.1 ± 0.7	2.5 ± 1.2	2.0 ± 0.6	1.5 ± 0.4*	2.2 ± 0.7
E deceleration time (ms)	171.6 ± 44.7	163.1 ± 32.9	140.5 ± 34.3*	174.8 ± 51.8	162.8 ± 31.3
***Systemic atrioventricular annular tissue velocities***					
s (cm/s)	5.6 ± 1.7*	5.9 ± 2.2*	5.0 ± 0.9*	3.7 ± 0.9*	7.9 ± 1.5
e (cm/s)	10.6 ± 3.1*	11.5 ± 2.6	5.4 ± 1.9*	5.9 ± 1.4*	12.1 ± 2.3
a (cm/s)	4.5 ± 1.9*	4.5 ± 1.5*	4.7 ± 1.8	4.2 ± 1.3*	5.7 ± 1.2
e/a ratio	2.8 ± 1.2*	2.9 ± 1.2*	1.4 ± 0.5*	1.6 ± 0.6*	2.2 ± 0.6
E/e ratio	9.3 ± 2.9	8.8 ± 3.2	18.5 ± 8.2*	10.4 ± 2.7*	8.3 ± 2.1
IVA (m/s^2^)	0.6 ± 0.3*	0.9 ± 0.6*	0.7 ± 0.4*	0.9 ± 0.4*	1.2 ± 0.4
***Subpulmonary atrioventricular annular tissue velocities***					
s (cm/s)	6.0 ± 1.5*	4.7 ± 1.3*	4.9 ± 1.3*	—	10.0 ± 1.6
e (cm/s)	7.8 ± 3.2*	8.9 ± 2.0*	7.2 ± 2.8*	—	11.9 ± 2.1
a (cm/s)	4.1 ± 1.9*	5.2 ± 1.1*	3.7 ± 0.9*	—	7.1 ± 1.7
e/a ratio	2.5 ± 1.9*	1.8 ± 0.5	2.1 ± 1.0	—	1.8 ± 0.5
IVA (m/s^2^)	0.4 ± 0.2*	1.0 ± 0.4*	0.9 ± 0.4*	—	1.8 ± 0.6
***Systemic ventricular longitudinal deformation***					
GLS (%)	13.5 ± 3.1*	13.0 ± 2.4*	12.1 ± 1.9*	12.1 ± 2.6*	17.4 ± 2.3
SRs (/s)	0.72 ± 0.17*	0.69 ± 0.14*	0.62 ± 0.12*	0.69 ± 0.11*	0.92 ± 0.15
SRe (/s)	1.00 ± 0.31*	1.16 ± 0.22*	0.80 ± 0.20*	0.81 ± 0.28*	1.53 ± 0.33
SRa (/s)	0.51 ± 0.17*	0.48 ± 0.12*	0.42 ± 0.14*	0.51 ± 0.21*	0.63 ± 0.15
***Systemic ventricular circumferential deformation***					
GCS (%)	16.7 ± 3.6	14.5 ± 4.2	13.5 ± 5.4	15.1 ± 3.0	16.3 ± 2.4
SRs (/s)	0.94 ± 0.20	0.92 ± 0.22	0.81 ± 0.23*	0.75 ± 0.22*	1.01 ± 0.21
SRe (/s)	1.65 ± 0.43*	1.32 ± 0.31	1.30 ± 0.71	1.20 ± 0.44	1.35 ± 0.31
SRa (/s)	0.33 ± 0.17*	0.37 ± 0.15	0.38 ± 0.23	0.41 ± 0.18	0.43 ± 0.17

Abbreviations: A, peak systemic atrioventricular inflow velocity at late diastole; a, late diastolic annular myocardial.

velocity; E, peak systemic atrioventricular inflow velocity at early diastole; e, early diastolic annular myocardial.

velocity; GCS, global circumferential strain; GLS, global longitudinal strain; IVA, myocardial isovolumic acceleration;

s, systolic annular myocardial velocity; SRa, late diastolic strain rate; SRe, early diastolic strain rate.

*p < 0.05 compared with controls.

Strain imaging revealed significantly lower global ventricular systolic longitudinal strain and strain rate and early and late diastolic strain rates in all four groups of patients compared with controls (all p < 0.001). On the other hand, the circumferential strain was similar between all patients and controls (all p > 0.05).

**Figure 2:**
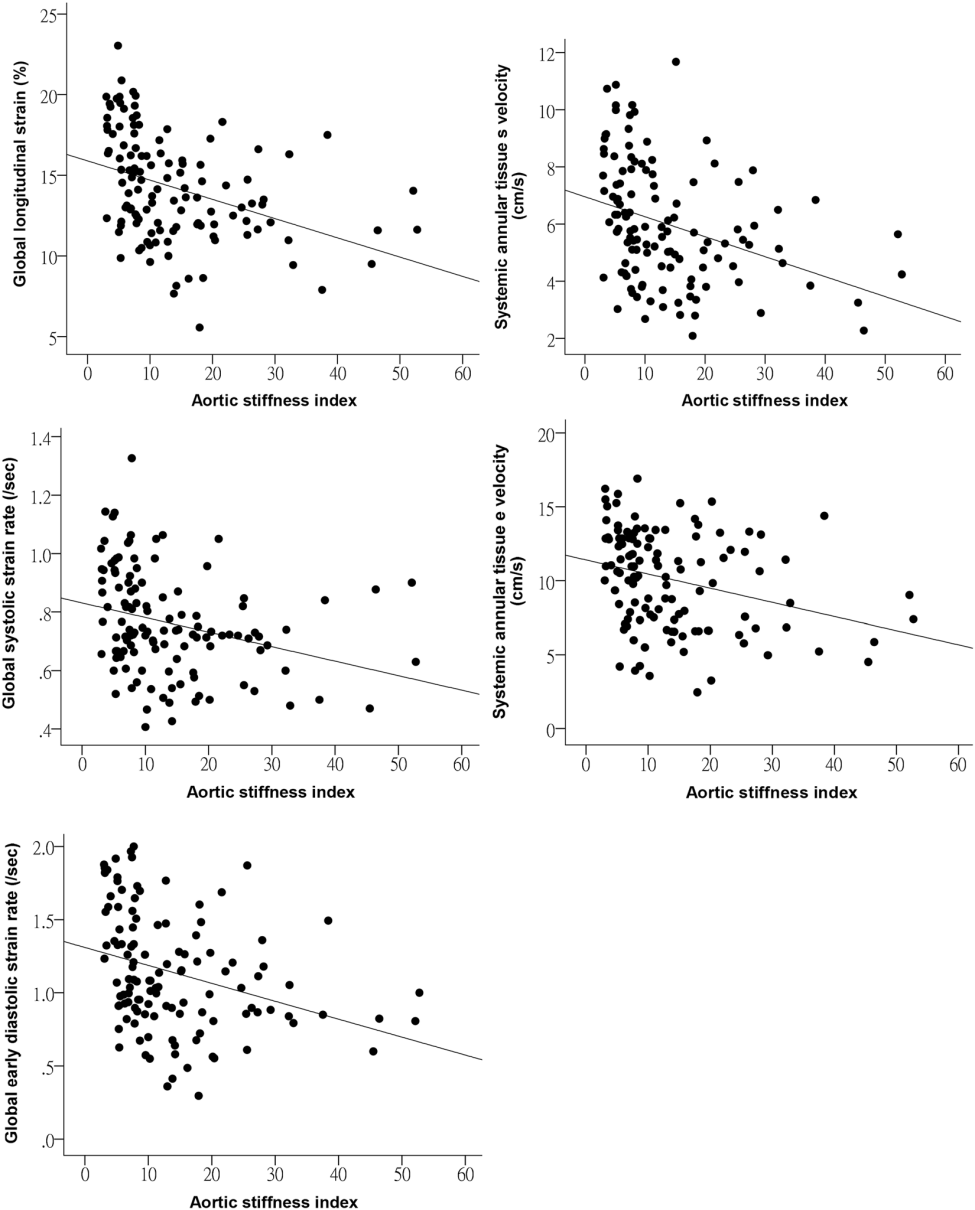
Scatter plots showing the relationships between ascending aortic stiffness index and indices of systemic ventricular deformation and systemic ventricular annular velocities.

For the entire cohort, aortic stiffness correlated negatively with systemic ventricular global longitudinal systolic strain (r = −0.37, p < 0.001), systolic strain rate (r = −0.29, p < 0.001), and early diastolic strain rate (r = −0.32, p < 0.001) (Fig. 2). Furthermore, aortic stiffness correlated negatively with systemic atrioventricular valvar *s* (r = −0.34, p < 0.001) and *e* (r = −0.30, p = 0.001) velocities.

### Circulating levels of TGFβ1, MMP2 and MMP9

The circulating of TGF-β1, MMP-2 and MMP-9 levels differed significantly among groups (ANOVA p < 0.001) (Table 4). Patients with repaired TOF had significantly higher circulating levels of TGF-β1 (p = 0.005), MMP-2 (p = 0.001) and MMP-9 (p < 0.001) than controls. Additionally, patients with TGA post atrial switch operation (p = 0.034) and those after Fontan operation (p < 0.001) had MMP-2 levels significantly greater than those of controls.

**Figure 3:**
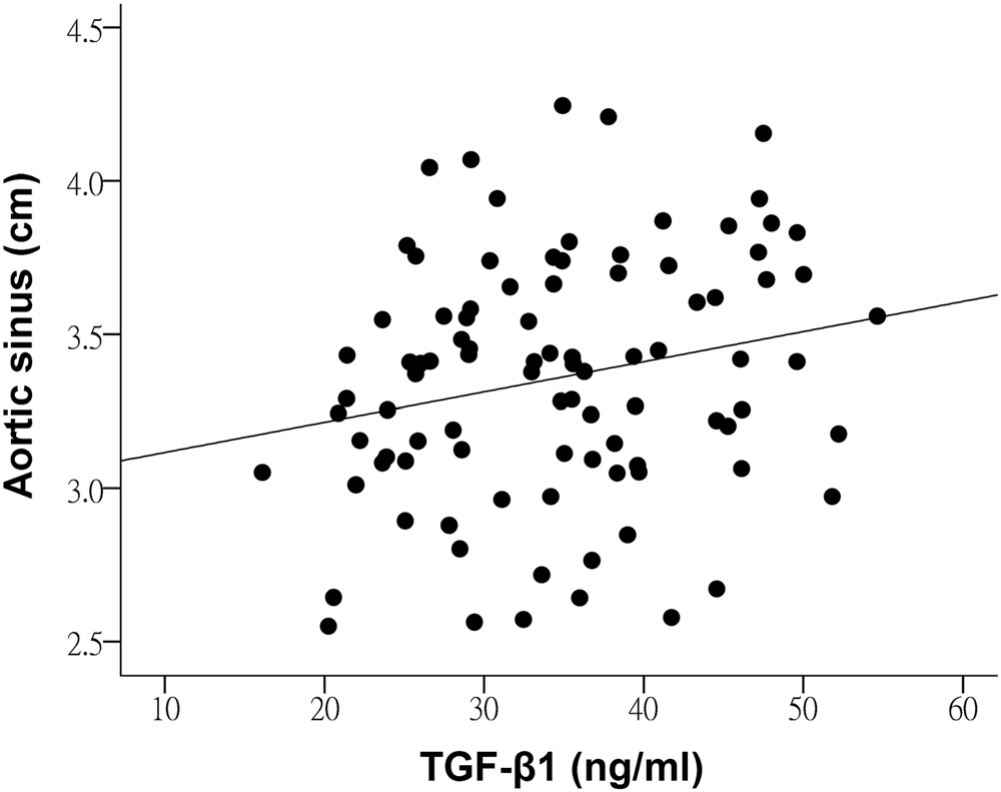
Scatter plot showing the relationships between circulating levels of TGF-β1 and aortic sinus dimension in patients.

In all patients, the circulating TGF-β1 levels correlated significantly with MMP-9 (r = 0.44, p < 0.001) but not MMP-2 (p > 0.05) levels. Furthermore, the circulating levels of TGF-β1 in patients as a group were found to correlate significantly, albeit weakly, with the size of the aortic sinus (r = 0.22, p = 0.035) (Fig. 3). There were no significant associations between circulating levels of TGF-β1, MMP-2, MMP-9 and indices of aortic elasticity (all p > 0.05).

Multivariate analysis of the entire cohort revealed that the diagnosis of TOF was an independent determinant (β = 0.34, p < 0.001) of circulating TGF-β1 levels after adjustment for other congenital heart disease categories, age, sex, aortic sinus dimension, and aortic stiffness index.

## Discussion

The main findings of the present study are (1) significant elevation of circulating levels of TGF-β1, MMP-2, and MMP-9 in patients with repaired TOF, (2) a positive correlation between circulating levels of TGF-β1 and MMP-9, and (3) an association, albeit relatively weak, between circulating TGF-β1 levels and the aortic sinus dimension in the four groups of congenital heart patients studied. Furthermore, increased aortic stiffness in the four groups of patients was found to be associated with greater aortic root dilation and worse systemic ventricular systolic and diastolic function. To our knowledge, this is the first study to explore circulating TGF-β1 and MMP levels in adolescents and young adults with congenital heart disease and their relationships with aortic structure and function.

Given the central role of TGF-β1 in vascular morphogenesis and extracellular matrix homeostasis, there is an increasing interest in the role of TGF-β1 in vascular remodeling in patients^17^. Alteration of TGF-β1 signaling has been shown in bicuspid aortopathy and recognized as a key component in the pathogenesis of thoracic aortic aneurysms^10^. Single nucleotide polymorphism of genes, including *TGF-β1*, involved in the maintenance of the extracellular matrix of the arterial wall has been associated with the risk of development of intracranial aneurysms^18^. In Marfan syndrome, deficient amount of fibrillin has been proposed to increase the release of sequestered TGF-β1 and hence its tissue activity in a mouse model^11^, although it remains unclear whether it is the structural deficiency or functional deficiency in fibrillar protein that triggers the pathogenesis of aortic aneurysms. Increased circulating levels of TGF-β1 have been found in the mouse model and in patients with Marfan syndrome^12^. In patients with Marfan syndrome, the circulating TGF-β1 levels are associated with aortic root dilation^12,13^.

Our novel finding of increased circulating TGF-β1 levels in patients with repaired TOF is hence intriguing, and may be of clinical relevance. In the ascending aorta of patients with TOF, tricuspid atresia and double-outlet right ventricle, Sun *et al*. described overexpression of TGF-β1 with concomitant moderate fragmentation of elastic fibres^14^. It is possible that leaching of the aortic tissue TGF-β1 into the circulation, as hypothesized in the mouse model of Marfan syndrome^12^, might have accounted for our findings in repaired TOF patients, although this remains speculative. While subgroup analysis showed mild elevation of TGF-β1 levels in patients after atrial switch operation and Fontan procedures, the increase was not statistically significant. Nonetheless, increased circulating MMP-2 levels were found to be increased in these three groups of patients, and increased MMP-9 levels was also found in repaired TOF patients (Table 4). Our finding of an association, albeit relatively weak, between circulating TGF-β1 levels and size of the aortic sinus in our congenital heart patients as a group may shed lights on the pathogenesis of aortopathy in congenital heart disease and therapeutic interventions.

**Table 4:** Circulating TGF-β1, MMP-2 and MMP-9 blood levelsl.

	Repaired TOF (n = = 46)	TGA post ASO (n = 21)	TGA post atrial switch (n = 15)	Fontan (n = 27)	Controls (n = 36)
TGF-β1 (ng/ml)	38.7 ± 9.1*	30.5 ± 10.1	34.4 ± 6.1	33.8 ± 10.9	32.6 ± 9.9
MMP-2 (ng/ml)	234.0 ± 41.5*	219.2 ± 37.8	227.1 ± 36.0*	261.7 ± 39.1*	204.3 ± 33.2
MMP-9 (ng/ml)	325.0 ± 151.0*	163.1 ± 97.3	197.5 ± 71.0	226.9 ± 193.4	196.3 ± 113.6

Abbreviations: MMP-2, matrix metalloproteinase 2; MMP-9, matrix metalloproteinase 9; TGF-β1, transforming growth factor- β1.

*p < 0.05 compared with controls.

Circulating TGF-β1 levels correlated with MMP-9 levels in our patients. Upregulation of MMP expression by TGF-β1 is well reported^19^. Although we failed to show significant associations between circulating levels of MMPs and aortic sinus dimensions in our patients as a group, increased MMP-2 and MMP-9 expression has been found in human tissues of abdominal aortic aneurysm^20^. In patients with severe aortic stenosis and ascending aortic dilation, circulating levels of MMP-2 and MMP-9 were found to be significantly elevated^21^. Indeed, the present study provides the first piece of evidence of elevated MMP-2 and MMP-9 in adolescents and adults with repaired congenital heart disease at risk of ascending aortopathy. Taken together, our data suggest possible associations between perturbation of the TGF-β1 signaling pathway and downstream MMP expression and development of aortopathy in patients with repaired TOF, and possibly in patients with TGA after atrial switch operation and Fontan patients. On the other hand, dilation of the sutured neo-aortic root, which originates from the pulmonary arterial sinus, in patients with complete TGA after arterial switch operation probably involves different mechanisms.

An evidence-based approach to the management of aortopathy in congenital heart disease is lacking^1–3^. Our findings suggest that tackling of the TGF-β1 signaling pathway and MMP activation may potentially be useful in the management of congenital heart disease-related aortopathy, in particular in patients with repaired TOF. Data on the use of angiotensin-converting enzyme inhibitor (ACEI)^22,23^ and angiotensin-receptor blocker (ARB)^11,24^ to slow progression of aortic root dilation through modulation of the TGF-β1 signaling pathway in Marfan syndrome are encouraging. Doxycycline, which inhibits MMPs^25^, has been shown to reduce the progression of abdominal aortic aneurysms in a mouse model of Marfan-associated aneurysm^26^. Taken together, our findings provide a basis for trials to explore the potential usefulness of ACEI, ARB, and doxycycline in patients after TOF repair, atrial switch operation, and Fontan procedure with aortopathy. Indeed, ACEI^27^ and ARB^28^ may further benefit our patients through reduction of the aortic stiffness. While we did not find associations between indices of aortic elastic properties and the circulating levels of TGF-β1 and MMPs in patients as a group, the adverse effects of arterial stiffening on aortic root dilation and ventricular dysfunction have been shown in the present and previous studies^29–31^.

Several limitations to this study require comments. First, this cross-sectional study cannot provide data on the trajectory of circulating levels of TGF-β1 and MMPs in all patients since surgery. Longitudinal studies are required to determine the prognostic value of these circulating markers and the effect of therapies that modulate the TGF-β1 signaling pathway or the activity of MMPs. Second, the relatively small number of patients in each of the patient cohorts might have limited the power to detect small differences in circulating TGF-β1 levels. However, the finding of increased MMPs in different patient groups, except for patients after arterial switch operation, supports the proposition of associations between alteration of TGF-β1 signaling and its downstream pathway and aortopathy. Nonetheless, the relatively weak correlation between TGF-β1 level and aortic sinus dimension suggests that other factors are also contributing to aortic root dilation in these congenital heart patients. A larger scale study is undoubtedly required to confirm our initial findings. Third, the origin of the circulating TGF-β1 and MMPs is unclear. Further studies are required to study the relationships between the expression of tissue TGF-β1 and MMP in the dilated aortic root of congenital heart patients and the corresponding circulating levels. Overlapping of TGF-β1 and MMP levels between all patients and controls may perhaps be related to the mild degree of aortic root dilation in our young patient cohorts. Serum MMP levels have been found to be higher than the corresponding plasma levels, related possibly to the release of MMPs from blood cell^32^. Nonetheless, the reported strong correlations between MMP-2 and -9 levels measured in serum and in plasma^33^ provide support on the validity of the interpretation of our findings. Fourth, echocardiographic imaging of the anterior retrosternal aorta in patients with TGA after atrial switch operation may be difficult. Finally, we did not assessment the lifestyle, physical activity levels, and cardiovascular risk factors in patients and controls. These factors are of relevance when interpreting the levels of circulating MMPs^34,35^ and should be included in subsequent studies.

In conclusion, increased circulating levels of TGF-β1, MMP-2, and MMP-9 are found in patients with repaired TOF, and increased circulating levels of MMP-2 are also evident in patients with complete TGA post atrial switch operation and in those with functional single ventricles after Fontan procedure. Trials of therapies that target at the TGF-β1 pathway and MMPs for the management of aortopathy in these congenital heart patients are warranted.

## Data Availability

Raw data are available upon request for research use.
